# Whole-genome sequencing reveals genomic characterization of *Listeria monocytogenes* from food in China

**DOI:** 10.3389/fmicb.2022.1049843

**Published:** 2023-01-16

**Authors:** Shunshi Ji, Zexuan Song, Lijuan Luo, Yiqian Wang, Lingling Li, Pan Mao, Changyun Ye, Yan Wang

**Affiliations:** ^1^State Key Laboratory of Infectious Disease Prevention and Control, National Institute for Communicable Disease Control and Prevention, Collaborative Innovation Center for Diagnosis and Treatment of Infectious Diseases, Chinese Center for Disease Control and Prevention, Beijing, China; ^2^School of Biotechnology and Biomolecular Sciences, University of New South Wales, Sydney, NSW, Australia

**Keywords:** *Listeria monocytogenes*, foodborne, whole-genome sequencing, genomic characterization, inlA PMSCs

## Abstract

**Introduction:**

*Listeria monocytogenes* is a foodborne bacterium that could persist in food and food processing environments for a long time. Understanding the population structure and genomic characterization of foodborne *L. monocytogenes* is essential for the prevention and control of listeriosis.

**Methods:**

A total of 322 foodborne *L. monocytogenes* isolates from 13 geographical locations and four food sources in China between 2000 and 2018 were selected for whole-genome sequencing.

**Results:**

*In silico* subtyping divided the 322 isolates into five serogroups, 35 sequence types (STs), 26 clonal complexes (CCs) and four lineages. Serogroup IIa was the most prevalent serogroup and ST9 was the most prevalent ST of foodborne *L. monocytogenes* strains isolated in China. The in-depth phylogenetic analysis on CC9 revealed that ST122 clone might be original from ST9 clone. Furthermore, 23 potentially relevant clusters were identified by pair-wised whole-genome single nucleotide polymorphism analysis, indicating that persistent- and/or cross-contamination had occurred in markets in China. ST8 and ST121 were the second and third top STs of *L. monocytogenes* in China, which had heterogeneity with that of *L. monocytogenes* isolates from other countries. The antibiotic resistance genes *aacA4*, *tetM*, *tetS*, *dfrG* carried by different mobile elements were found in *L. monocytogenes* strains. One lineage II strain carrying Listeria Pathogenicity Island 3 was first reported. In addition, a novel type of premature stop codon in *inlA* gene was identified in this study.

**Discussion:**

These findings revealed the genomic characteristics and evolutionary relationship of foodborne *L. monocytogenes* in China on a scale larger than previous studies, which further confirmed that whole-genome sequencing analysis would be a helpful tool for routine surveillance and source-tracing investigation.

## Introduction

*Listeria monocytogenes*, as an important foodborne pathogen, is the causative agent of human listeriosis with clinical symptoms ranging from self-limiting gastroenteritis to severe invasive infections (Swaminathan and Gerner-Smidt, 2007). It has a significant impact on public health around the world due to high mortality rate in hospitalized patients (Schlech III, 2019). Immunocompromised individuals, pregnant women, neonates and the elderly are often at high risk for the aggressive forms of listeriosis (Wilking et al., 2021). *L. monocytogenes* is widely distributed in nature and more commonly found in a variety of food products and food-associated environments (Wang et al., 2019). This bacterium can contaminate food at any time from production to consumption. It can also shed into the environment, colonize and multiply, and then cause persistent contamination and/or cross-contamination. A number of research have shown that epidemic and sporadic listeriosis were often associated with consumption of *L. monocytogenes* contaminated foods (McLauchlin et al., 2021a,b). Food contamination caused by *L. monocytogenes* poses a serious threat to food safety and public health worldwide.

*L. monocytogenes* has evolved into four lineages, lineage I to lineage IV. Lineage I, including serotypes 1/2b, 3b, 4b, 4d, 4e, and 7 occurs frequently among human cases of listeriosis. While lineage II including serotypes 1/2a, 3a, 1/2c, and 3c is overrepresented in food products and food-associated environments. Lineage III and IV, corresponding serotypes 4a, 4c, and 4ab, are rare and commonly isolated from animal sources (Leclercq et al., 2011). Doumith et al. developed a multiplex PCR method to classify *L. monocytogenes* strains into five serogroups by five marker genes (*lmo0737*, *lmo1118*, *ORF2819*, *ORF2100* and *prs*), namely serogroups IIa (serotypes 1/2a and 3a), IIb (serotypes 1/2b, 3b, and 7), IIc (serotypes 1/2c and 3c), IVb (serotypes 4b, 4d, and 4e) and L (4a, 4ab, and 4c; Doumith et al., 2004, 2005). Multi-locus sequence typing (MLST) remains a practical and portable method to meet the data exchange demands of different laboratories around the world (Salcedo et al., 2003). MLST can divide *L. monocytogenes* isolates into large number of sequence types (STs), which can further be grouped into serval clonal complexes (CCs). A CC of *L. monocytogenes* is defined as a group of STs, where each ST shares six of seven identical alleles with one other ST in the group (Salcedo et al., 2003). CC1, CC2, CC4, and CC6 were hypervirulent clones and strongly associated with listeriosis outbreaks. In contrast, CC9 and CC121 were hypovirulent clones and overrepresented in food sources (Maury et al., 2019).

However, traditional molecular subtyping methods, such as MLST and pulsed-field gel electrophoresis (PFGE), have insufficient discriminative power for source-trace investigations of *L. monocytogenes* contamination (Moura et al., 2016). The main reason for this is the conserved genomic backbone and the limited resolution of the technologies used (Moura et al., 2016). Whole-genome sequencing (WGS) has been widely used in the investigation of listeriosis outbreaks for a decade, for example, it facilitated to identify ST6 *L. monocytogenes* as the causative agent of the listeriosis outbreak in South Africa and guided the traceback investigations (Thomas et al., 2020). Furthermore, WGS analysis is of great significance for both biological and epidemiological studies on *L. monocytogenes*, through which we can obtain more details of molecular characteristics and refine the genetic relationship among them. *L. monocytogenes* genome-based typing and population biology study at the national level is lacking in China, although related projects have been carried out in part of local regions. A 10-year surveillance of *L. monocytogenes* in Shanghai found CC87 isolates persist in different food groups (Zhang et al., 2020). Another study found *L. monocytogenes* isolated from pork in Wuhan differed by few SNPs indicated a common supply source contamination (Wang et al., 2021). However, there are no reports analyzed isolates from the whole country or multiple provinces in China. Here, we sequenced 322 *L. monocytogenes* isolates from 13 different provinces/cities across China to determine (i) the population structure and diversity of *L. monocytogenes* from food in China based on whole-genome sequencing, (ii) the distribution of the predominant clones in the global context of corresponding clones, and (iii) the genetic characteristics, including virulence factors, stress survival genes and antibiotic resistance genes, of food-source *L. monocytogenes* in China.

## Materials and methods

### Selection of *Listeria monocytogenes* isolates in this study and public genomes for comparison

A total of 322 foodborne *L. monocytogenes* isolates from 12 provinces and one municipality in China between 2000 and 2018 were chosen in this study. These strains were isolated from four types of food including raw meat (*n* = 107), raw poultry (*n* = 81), ready-to-eat (RTE) food (*n* = 44), and aquatic products (*n* = 31). There were also 59 isolates recorded as unknown food sources. Details of each isolate were listed in Supplementary Table S1.

To examine the relationship between the isolates of the prevalent subtypes (ST9, ST8, ST121, and ST87) in China and the isolates from other countries, a total of 310 genomes available from GenBank were selected for comparative analysis. These genomes included 79 isolates from China and 231 isolates from 17 other countries, and the detailed information was listed in Supplementary Table S2.

### DNA extraction and whole-genome sequencing

All *L. monocytogenes* isolates were streaked on Brain Heart Infusion agar plates and incubated at 37°C for 18 h. Genomic DNA was extracted using Wizard® Genomic DNA Purification Kit (Promega, United States) according to the instructions of the manufacturer for Gram-positive bacteria. DNA quality and concentration were determined using NanoDrop Spectrophotometer (Thermo Scientific, United States). WGS was performed on Illumina HiSeq X PE150 platform by Novegene (Beijing, China), with a coverage rate of more than 100-fold. Low-quality reads, ambiguous sequences and adapter sequences were filtered using FastQC. Then, SOAP *de novo* v2.04 was used to assemble genome sequence. Whole genome annotation was performed using Prokka pipeline v1.14.6.

### *In silico* subtyping and phylogenetic analysis

Serogroup identification and MLST analysis were performed *in silico* on BIGSdb-Lm platform.^1^ Snippy pipeline v4.6.0 was used to generate core genome SNPs by mapping genome sequences with the reference genome sequence EGD-e (NZ_CP023861.1). The recombination was removed by Gubbins pipeline, whole-genome phylogeny of strains was inferred by FastTree. Then the core-SNPs phylogenetic tree was visualized and edited by online software iTol^2^ and Phandango^3^ (Hadfield et al., 2018; Letunic and Bork, 2021). Roary v3.13.0 was used to define the pan genome of strains using the outputs of Prokka annotation. Pan-genome were classified according to the principles of previously reported (Page et al., 2015). The phylogenetic tree for pan-genome was visualized using online software Phandango.

### Identification of genes for stress resistance, virulence factors and antibiotic resistance

According to BIGSdb-Lm database, BLASTN algorithm was used to detect genes encoding stress resistance, virulence factors and antibiotic resistance, with the minimum of 80% coverage and 80% identity (Camargo et al., 2019).

## Results

### Characteristics of whole genome sequencing

The high-quality reads of each isolate with an average coverage above 100X were assembled to draft genome sequences. The genome assemblies contained seven to 48 scaffolds with the sizes ranging from 2.8 Mbp to 3.2 Mbp and the GC content about 37.87%. There were 2,748 to 3,243 protein-coding sequences predicted and annotated in the genomes in this study (Supplementary Table S3).

### Serogroup identification and MLST *in silico*

**Figure 1:**
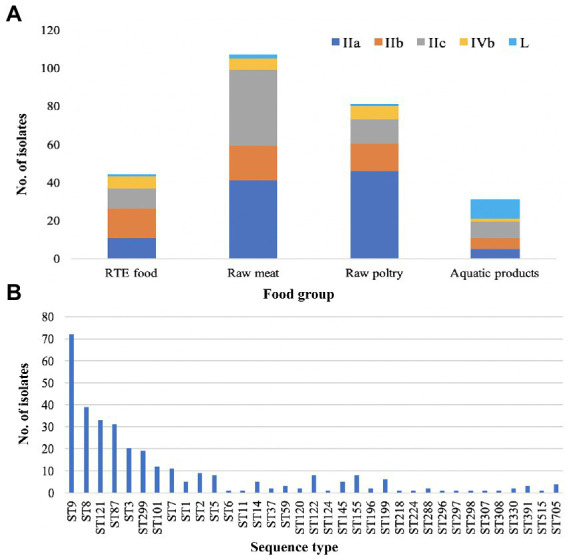
Distribution of *Listeria monocytogenes*. **(A)** Distribution of five serogroups (IIa, IIb, IIc, IVb and L) in four main food groups. **(B)** Distribution of sequence types (STs) in the 322 *L. monocytogenes* isolates.

Five serogroups were identified among 322 *L. monocytogenes* isolates based on five genes (*lmo0737*, *lmo1118*, *ORF2110*, *ORF2819*, and *prs*), including serogroup IIa (*n* = 131, 40.68%), IIb (*n* = 68, 21.12%), IIc (*n* = 80, 24.84%), IVb (*n* = 24, 7.45%), and L (*n* = 19, 5.90%). Serogroup distributions of *L. monocytogenes* isolates were different in four food types (Figure 1A). Serogroups IIa, IIb, IIc and IVb accounted for a similar proportion of isolates from RTE food. Serogroup IIa and IIc isolates were predominant in raw meat with the frequency of 38.31 and 37.38%, respectively. Serogroup IIa isolates were overrepresented in raw poultry with a frequency of 56.78%. For aquatic products, serogroup L was more common with a frequency of 32.26% than other four serogroups.

Based on seven-genes MLST (Salcedo et al., 2003), all the studied isolates were classified into 35 STs which were further grouped into 26 CCs. ST9 was the predominant ST (*n* = 72, 22.36%), followed by ST8 (*n* = 39, 12.11%), ST121 (*n* = 33, 10.25%), ST87 (*n* = 31, 9.63%), ST3 (*n* = 20, 6.21%), ST299 (*n* = 19, 5.90%), ST101 (*n* = 12, 3.73%), and ST7 (*n* = 11, 3.42%). The remaining 27 STs contained less than 10 isolates for each (Figure 1B). Overall, 28.57, 65.53 and 5.90% of isolates were assigned to lineage I, lineage II and lineage III, respectively.

### Core-SNP phylogenetic analysis

**Figure 2:**
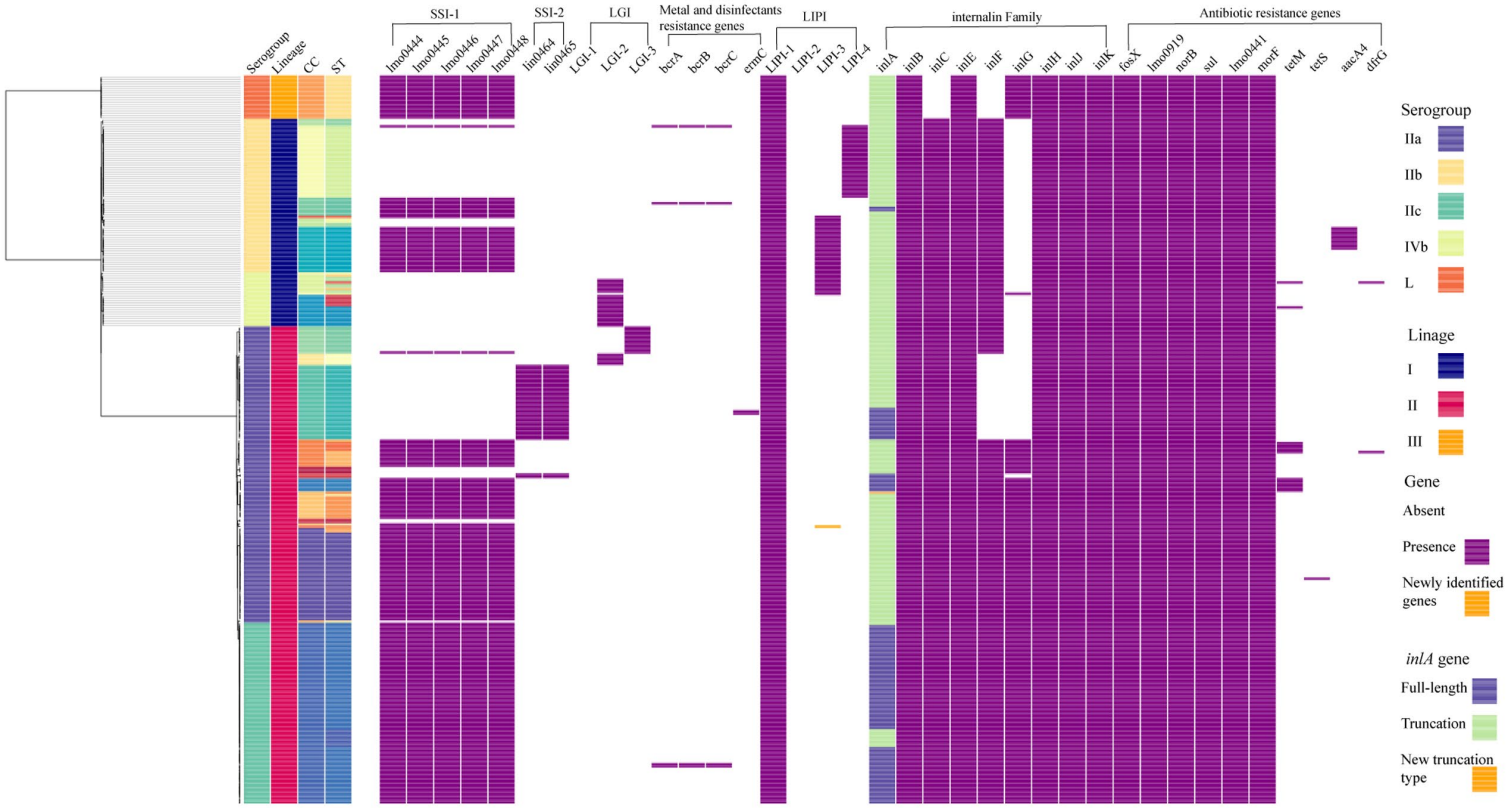
The core-SNP phylogenetic tree and genomic characterization of 322 foodborne *L. monocytogenes* isolates from China. Maximum likelihood phylogeny of 322 *L. monocytogenes genomes based* on 16,383 core-SNPs with EGD-e as the reference. Information of serogroup (dark purple boxes represent serogroup IIa, light orange boxes represent serogroup IIb, blue-green boxes represent serogroup IIc, light green boxes represent serogroup IVb, dark orange boxes represent serogroup L), lineage (dark blue boxes represent lineage I, rose red boxes represent lineage II, orange boxes represent lineage III), clonal complex and sequence type (they were clustered by different colors) are provided on the right. The distribution of different genes in *L. monocytogenes* was visualized using Phandango. The presence of a gene was marked with a purple box. The newly identified genes were marked with an orange box. For *inlA* gene, light green boxes represent full-length *inlA* gene, dark blue boxes represent truncated *inlA* gene.

A maximum-likelihood phylogenetic tree was constructed based on the alignment of 16,383 core-SNPs that were identified by Snippy (Figure 2). Three clusters corresponding three lineages were displayed. In addition, the phylogenetic tree also showed that the genomes clustered based on CCs. Notably, all ST122 isolates belong to a sub-clade located within the clade of ST9, not next to the clade of ST9 (Supplementary Figure S1A). To clarify the refine relationship of ST9 and ST122, the phylogenetic analysis was performed based on the SNPs among genomes of the two STs isolates in this study and four ST122 genomes from public database (Accession numbers: GCA_002114825.1, GCA_002114845.1, GCA_002250305.1, and GCA_009664775.1; Supplementary Figure S1B). Another tree was constructed based on the binary presence and absence of accessory genes when the analysis of pan-genome was performed using Roary (Supplementary Figure S1C). Both the trees showed that a subset of ST9 isolates were phylogenetically closer to ST122 isolates than the other ST9 isolates, in other words, these ST122 isolates had a recent common ancestor with a subset of ST9 isolates.

### Pair-wised wgSNP analysis of closely related isolates

Based on the above phylogenetic analysis, there were 87 core-SNP profiles represented by more than one isolates. To zoom in the differences among isolates within each profile, pair-wised SNP analysis was performed by Snippy separately. The numbers of SNP differences among isolates were minimal suggesting potential epidemiological links (Blanc et al., 2020). Twenty-three potentially relevant clusters were ≤ 12 pair-wised SNPs (Zhang et al., 2021; Table 1). These potential related isolates assigned into 10 STs with the top three STs of ST9, ST8, and ST299. Among these possible contamination events, eight clusters (Cluster 1, 3, 4, 7, 9, 10, 21, and 22) were caused by the isolates from different sources (market, farm or restaurant) in the same province in the same year, six clusters (Cluster 5, 6, 13, 17,19, and 23) were caused by the isolates from different food products from the same market in the same year, five clusters (Cluster 2, 8, 11, 12, and 14) were caused by the isolates from different sources in the same province in different years, two clusters (Cluster 15 and 20) were caused by the isolates from the same market in different years and two clusters (Cluster 16 and 18) were caused by the isolates from different provinces in different years.

**Table 1:** Strains used for pair-wised wgSNP analysis.

Cluster	ST	Isolate ID	Time	Province^a^	Market^b^	Source	Food group	Number of SNP^c^
1	ST9	ICDC_LM3007	2017	YN	M1	Minced pork	Raw meat	5
	ST9	ICDC_LM3008	2017	YN	M2	Cooked meat products	RTE Food	R
2	ST9	ICDC_LM172	2008	AH	M3	Beef	Raw meat	7
	ST9	ICDC_LM176	2010	AH	M4	Fish	Aquatic products	R
3	ST9	ICDC_LM265	2010	AH	M5	Duck	Raw poultry	3
	ST9	ICDC_LM266	2010	AH	M6	Chicken	Raw poultry	R
4	ST9	ICDC_LM340	2011	AH	M5	Pork	Raw meat	5
	ST9	ICDC_LM426	2011	AH	R1	Cooked meat products	RTE Food	R
5	ST9	ICDC_LM316	2009	ZJ	M7	Pork	Raw meat	1
	ST9	ICDC_LM318	2009	ZJ	M7	Beef	Raw meat	R
6	ST9	ICDC_LM321	2009	ZJ	M8	Chicken	Raw poultry	4
	ST9	ICDC_LM328	2009	ZJ	M8	Fish	Aquatic products	R
7	ST9	ICDC_LM180	2008	AH	M9	Fish	Aquatic products	4
	ST9	ICDC_LM184	2008	AH	M10	Fish	Aquatic products	R
8	ST8	ICDC_LM187	2008	AH	M5	Rabbit	Raw meat	11
	ST8	ICDC_LM267	2010	AH	M11	Chicken	Raw poultry	R
9	ST8	ICDC_LM480	2012	AH	M12	Beef	Raw meat	0
	ST8	ICDC_LM481	2012	AH	M13	Beef	Raw meat	R
10	ST8	ICDC_LM288	2007	ZJ	M14	Pork	Raw meat	8
	ST8	ICDC_LM300	2007	ZJ	M15	Chicken	Raw poultry	R
11	ST8	ICDC_LM2659	2009	AH	M9	Chicken	Raw poultry	0
	ST8	ICDC_LM2661	2013	AH	F1	Fish	Aquatic products	R
12	ST8	ICDC_LM186	2008	AH	M13	Chicken	Raw poultry	R
	ST8	ICDC_LM2640	2009	AH	R2	Cooked meat products	RTE Food	0
	ST8	ICDC_LM2641	2009	AH	R2	Cooked meat products	RTE Food	0
13	ST8	ICDC_LM320	2009	ZJ	M8	Chicken	Raw poultry	4
	ST8	ICDC_LM327	2009	ZJ	M8	Fish	Aquatic products	R
14	ST155	ICDC_LM292	2007	ZJ	M14	Pork	Raw meat	6
	ST155	ICDC_LM304	2008	ZJ	M16	Chicken	Raw poultry	R
15	ST705	ICDC_LM291	2007	ZJ	M14	Pork	Raw meat	2
	ST705	ICDC_LM303	2008	ZJ	M14	Pork	Raw meat	R
16	ST101	ICDC_LM114	2005	ZJ	M17	Chicken	Raw meat	1
	ST101	ICDC_LM179	2008	AH	F2	Crayfish	Aquatic products	R
17	ST121	ICDC_LM286	2007	ZJ	M14	Pork	Raw meat	1
	ST121	ICDC_LM297	2007	ZJ	M14	Beef	Raw meat	R
18	ST299	ICDC_LM561	2006	NX	M18	Fish	Aquatic products	5
	ST299	ICDC_LM2644	2009	AH	M6	Fish	Aquatic products	R
19	ST299	ICDC_LM190	2008	AH	M3	Beef	Raw meat	9
	ST299	ICDC_LM191	2008	AH	M3	Fish	Aquatic products	R
20	ST299	ICDC_LM192	2008	AH	M3	Chicken	Raw poultry	2
	ST299	ICDC_LM197	2009	AH	M3	Egg	Eggshell	R
21	ST3	ICDC_LM272	2010	AH	M19	Pork	Raw meat	R
	ST3	ICDC_LM274	2010	AH	M5	Fish	Aquatic products	7
	ST3	ICDC_LM275	2010	AH	M6	Chicken	Raw poultry	7
	ST3	ICDC_LM278	2010	AH	M20	Jellyfish	Aquatic products	4
22	ST145	ICDC_LM594	2014	AH	F3	Chicken	Raw poultry	R
	ST145	ICDC_LM595	2014	AH	F4	Fish	Aquatic products	1
	ST145	ICDC_LM596	2014	AH	F5	Chicken	Raw poultry	0
	ST145	ICDC_LM597	2014	AH	F6	Pork	Raw meat	0
	ST145	ICDC_LM2660	2013	AH	M13	Cooked meat products	RTE Food	2
23	ST87	ICDC_LM3033	2018	YN	M1	Chicken	Raw poultry	R
	ST87	ICDC_LM3034	2018	YN	M1	Cooked meat products	RTE Food	6
	ST87	ICDC_LM3035	2018	YN	M1	Cooked meat products	RTE Food	3

Province^a^: YN, YunNan; ZJ, ZheJiang; AH, AnHui; NX, NingXia. Market^b^: M, Market; R, restaurant; F, Farm. RTE food: read-to-eat food. Number of SNP^c^: R, reference strain for each cluster of pair-wised SNP analysis

### The distribution of the prevalent subtypes of *Listeria monocytogenes* isolates from food in China in the context of global isolates

**Figure 3:**
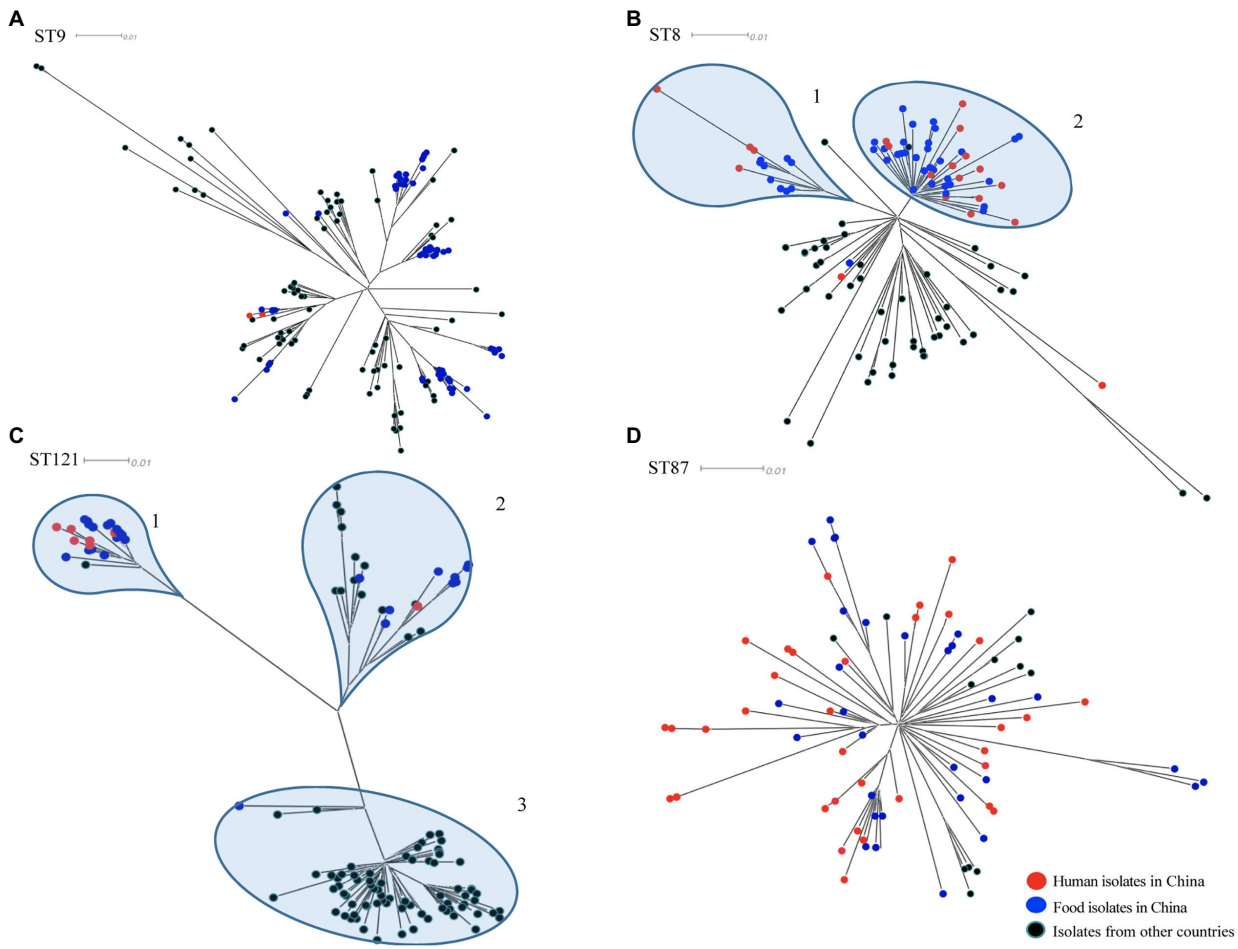
The core-SNP phylogenetic tree of prevalent subtypes of *L. monocytogenes* in China in the context of global isolates. The trees were visualized using SplitsTree 5. **(A)** Maximum likelihood phylogeny of 156 *L. monocytogenes* ST9 genomes based on 2,269 core-SNPs, annotation of SLCC2479 was used as reference. **(B)** Maximum likelihood phylogeny of 115 *L. monocytogenes* ST8 genomes based on 1,327 core-SNPs, annotation of ICDC_LM2 was used as reference. **(C)** Maximum likelihood phylogeny of 132 *L. monocytogenes* ST121 genomes based on 2,236 core-SNPs, annotation of ICDC_LM135 was used as reference. **(D)** Maximum likelihood phylogeny of 82 *L. monocytogenes* ST87 genomes based on 1,058 core-SNPs, annotation of ICDC_LM188 was used as reference. Human isolates in China are represented by red circles, food isolates in China are represented by blue circles, isolates from other countries are represented by black circles. Light blue circles represent clusters.

To determine the relationship between the top four prevalent STs from China (ST9, ST8, ST121, and ST87) and those from other countries, a total of 310 publically available genomes of *L. monocytogenes* were selected for comparative analysis. Here we also selected some isolates of each four ST from human cases in China to diversify the sources of isolates. For each ST, phylogenetic trees were separately constructed based on the alignment of the core-SNPs of isolates (Figure 3). The ST9 *L. monocytogenes* from China containing two isolates from clinical cases formed different clusters and were distributed across the phylogenetic tree of the global isolates (Figure 3A). The ST9 isolates in China were isolated from different regions and isolation years were clustered on the same branches, no epidemiological specific clustering was observed. For ST8, with exception of three outliers (one food-original and two clinical isolates), the ST8 isolates in China were grouped into two clusters (Figure 3B), and no evident temporal and spatial specificity were observed for the isolates of each cluster. For ST121, almost all isolates in China were distributed in the two out of three clusters, with exception of one food-original isolate that belonged to an international cluster. While 83.33% isolates of ST121 from other countries were grouped into the remaining cluster that was distinct from the clusters of strains isolated in China (Figure 3C). For ST87, isolates of ST87 from China and other countries were mixed with no epidemiological specific clusters observed (Figure 3D).

### Stress resistance genes in the studied *Listeria monocytogenes* isolates

In this study, all 322 *L. monocytogenes* isolates were screened for the presence of stress adaptation associated genes or gene clusters, including stress survival islet 1 (SSI-1), stress survival islet 2 (SSI-2), three *Listeria* genomic islands (LGI1, LGI2, and LGI3), two benzalkonium chloride resistance determinants *bcrABC* gene cassette and the *ermC* gene. The SSI-1 was detected in 203 isolates (63.04%), including 32.61% (30/92) of lineage I isolates, 72.64% (154/212) of lineage II isolates and all the lineage III isolates. The SSI-2 was observed only in ST121 and ST196 isolates of lineage II (Figure 2). None of the studied isolates harbored LGI-1. There were 25 isolates harbored LGI-2 that includes genes putatively involved in arsenic and cadmium resistance. All CC2 and ST14 isolates carried LGI-2 consisting of 34 genes, which is consistent with the firstly reported strain Scott A (Kuenne et al., 2013). In addition, all the six CC1 isolates harbored a variant of LGI-2 previously reported by Gray et al. (2021), which carried an additional *metC* homology gene. A variant of LGI-3 was observed in all ST101 isolates in this study, lacking four genes compared to the first reported LGI-3 in ST101 isolates by Palma et al. (2020). These four genes encode the transposase (*Tn3*), recombinase (*hin*), and Cd-resistance (*cadC* and *cadA1*), the locus-tag in strain A37-02-LmUB3PA: LmUB3PA_1699 - LmUB3PA_1702. *The bcrABC* gene cassette was observed in two isolates (each for ST5 and ST296) of lineage I, and two ST9 isolates of lineage II. Two ST121 isolates harbored the *ermC* gene (Figure 2).

### Virulence factors in the studied *Listeria monocytogenes* isolates

Three *L. monocytogenes*-specific pathogenicity islands (LIPI), LIPI-1, LIPI-3, and LIPI-4, were screened among the 322 foodborne isolates. LIPI-1 was highly conserved in all isolates. LIPI-3 was identified in the isolates belonging to a subset of lineage I isolates, including CC1, CC3, CC6, CC11, CC218, CC224, and CC288. Notably, one CC11/ST11 isolate ICDC_LM277 of lineage II carried the intact LIPI-3, a gene of it had 100% coverage and 96.937% identity with previously reported *llsH* gene in lineage I. LIPI-4 was identified in all ST87 (31/31) and ST296 (1/1) isolates belonging to lineage I.

**Figure 4:**
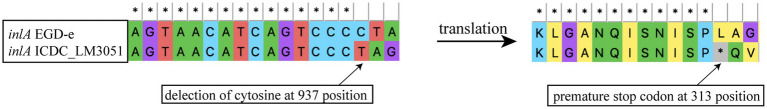
Newly identified *inlA* PMSC mutation type in *L. monocytogenes* as compared to full-length *inlA* gene from strain EGD-e.

The intact *inlA* gene encoding full-length InternalinA (InlA) was observed in 226 *L. monocytogenes* isolates, including 97.83% lineage I isolates, 55.45% lineage II isolates and 100% lineage III isolates in this study (Figure 2). The truncated *inlA* caused by premature stop codon (PMSC) mutations were observed in two ST5 isolates of lineage I, and 94 (44.55%) lineage II isolates belonging to ST7 (1/11), ST9 (71/72), ST121 (14/33), ST196 (2/2) and ST199 (6/6). Nine PMSC mutation types of *inlA* were identified, one of which was firstly reported here. The novel PMSC type existed in one ST7 isolate, a truncated InlA with a shorter peptide of 312 amino acids was caused by the deletion of cytosine in position 937 (Figure 4). For the other internalin genes, *inlB*, *inlE*, *inlH*, *inlJ* and *inlK* were found in all studied isolates. The *inlC* gene was absent in all ST299 isolates, while the *inlF* gene was absent in all ST14, ST121, and ST299 isolates. In addition, the *inlG* gene was absent in all lineage I isolates except for one ST6 isolate, and a subset of lineage II isolates which belonged to ST14, ST101, ST121, and ST196.

### Antibiotic resistance genes in the studied *Listeria monocytogenes* isolates

Ten antibiotic resistance genes were identified in this study (Figure 2). Six intrinsic antibiotic resistance genes, including *fosX* (resistance to fosfomycin), *lmo0441* (cephalosporin), *lmo0919* (lincosamides), *norB* (quinolones), *mprF* (cationic antimicrobial peptides) and *sul* (sulfonamides), were present in all studied isolates. Four acquired antibiotic resistance genes were identified in a small portion of the isolates. The aminoglycosides resistance gene *aacA4* was identified in 50% (10/20) of ST3 isolates, locating in a 57-kb prophage that was inserted between the *fosX* (locus tag in EGD-e: CRH04_RS08765) and *rlmD* (locus tag in EGD-e: CRH04_RS08770) genes. The *aacA4* gene positive isolates were found in three provinces in three different years, and they were isolated from four food sources, including RTE food, raw meat, raw poultry and aquatic products. The tetracycline resistance gene *tetS* was identified in one strain ICDC_LM3005 and was carried by a plasmid with a size of about 78 kb. Another tetracycline resistance gene *tetM* was observed in 13 isolates belonging to ST199 (*n* = 6), ST705 (*n* = 4), ST2 (*n* = 1), ST155 (*n* = 1), and ST515 (*n* = 1). The *tetM* gene from the ST199 isolates were carried by a transposon that was similar to Tn5801_B15 in *Enterococcus faecalis* with 100% coverage and 99.99% identity. The transposon harboring *tetM* gene was found in all ST199 isolates in this study, which were isolated from four provinces and three food sources. While the *tetM* gene from the ST705, ST2, and ST515 isolates were carried by a transposon that was similar to Tn916 in *Bacillus subtilis* with 100% coverage and 99.83% identity. Isolates carrying the Tn916 were distributed in three provinces and were isolated from three food sources. In addition, the *tetM* gene from the ST155 isolate was carried by a transposon Tn6198 that also carried the trimethoprim resistance gene *dfrG*. The *tetM* positive ST515 isolate in this study also carried *dfrG*, which was located downstream of a *tRNA-Val* gene along with two hypothetic proteins.

## Discussion

As a foodborne pathogen, *L. monocytogenes* can survive and proliferate in different kinds of food and food-associated environments, even for a long time (Fagerlund et al., 2020; Wieczorek et al., 2020). Since 2000, the national surveillance of *L. monocytogenes* in different food products in China has been established. Several studies have been conducted to investigate the population structure and molecular epidemiology of *L. monocytogenes* in China using molecular subtyping methods, such as PFGE and MLST (Hua et al., 2018; Wang et al., 2018). Genome-wide genotyping has been used to study the biodiversity of *L. monocytogenes* and the genetic relationships between isolates, but is limited to local areas in China. Here, we performed whole-genome sequencing and genetic characterization of 322 *L. monocytogenes* isolated from food products from 13 regions between 2000 and 2018 to provide insight into the genomic diversity, molecular characteristics, and phylogenetic relationships of *L. monocytogenes* in China.

As previously reported, serogroup IIa was the most frequently isolated serogroup in meat products and environmental surfaces (Centorotola et al., 2021). In this study, by subtyping *in silico*, serogroup IIa and ST9 were the most common subtypes of food-sourced *L. monocytogenes* in China, which was consistent with previous studies (Wang et al., 2012; Zhang et al., 2020). ST9 was a common clone of *L. monocytogenes* isolated worldwide from food especially from meat products (Moura et al., 2016). The second and the third top of STs (ST8 and ST121) in this study also belonged to serogroup IIa. All the serogroup IIc isolates belonged to CC9 including ST9 and ST122, and showed limited diversity of genomes compare to serogroup IIa, which comprised of multiple CCs. ST8 was the second prevalent ST, which was proposed as the subtype of epidemic clone 5 (ECV), and was the predominant ST responsible for the human listeriosis in Canada during 1988 and 2010 (Fagerlund et al., 2016). Previous studies have shown that ST8 *L. monocytogenes* is commonly found in both food products and human listeriosis in China (Li et al., 2018; Zhang et al., 2021). Full-length *inlA* and SSI-1 were carried by all ST8 isolates, which partially supported its full pathogenic potential and increasing tolerance of stress conditions. Therefore, more attention should be paid to ST8 *L. monocytogenes* in the surveillance in China. ST121 was identified as the third prevalent subtype of isolates from food in China. A number of genetic determinants had been identified to involved in survival and adaptation of ST121 strains in food and food associated environments, like SSI-2, the transposon Tn6188 and a high proportion of plasmids (Schmitz-Esser et al., 2021). Consistent with our previous study, ST87 had been always identified as a concerned subtype among the *L. monocytogenes* from diverse sources in China (Wang et al., 2019). And the recent study had revealed that several genetic elements, such as LIPI-4 and a conserved plasmid pLM1686, may contributed to its pathogenic potential and adaptation to harsh environment (Wang et al., 2019).

So far, no listeriosis outbreak of any scale has been reported in China, partly due to the different consumption patterns and food-handling habits. However, RTE food particularly Chinese cold dishes which were reported to increase the risk of infection by 3.43-fold (Niu et al., 2022). In this study, 15 STs isolates were detected from RTE food, some STs of them were reported to have a strong link to the outbreak and sporadic listeriosis around the world, such as ST1, ST2, ST5, ST8, and ST87 (Chen et al., 2018; Maury et al., 2019; Wang et al., 2019; Zhang et al., 2021). Therefore, it is very important to strengthen hygiene monitoring and disinfection of RTE food processing and storage environment.

Pair-wised SNPs detection was performed on the indistinguishable isolates by core-SNPs analysis based on the species of *L. monocytogenes*. Here, we found 23 potentially associated events related to *L. monocytogenes* contamination. Combining with the meta-data including isolation sources (market, restaurant or farm), isolation years and isolation regions, it suggested that food safety risks exist in multiple contamination scenarios, including long-term persistent contamination over time, cross-contamination in markets, and the spread of a single source to different markets. However, caution should be exercised in inferring relationships between isolates with closely related genomes from different provinces and over time, unless clear epidemiological information is available.

Core-SNPs analysis based on the species of *L. monocytogenes* here apparently did not provide sufficient resolution to differentiate between isolates within a given CC/ST. ST122 was a member of CC9 with a different allele of *ldh* gene (the allele number of *ldh* is four in ST9 and 62 in ST122). Deeper phylogenetic analysis showed that the emerging ST122 clone might diverge from the ST9 clone for its most recent ancestor sharing with a subset of ST9’s. It suggested that the population of CC9 *L. monocytogenes* may be undergoing a diversification process.

In-depth phylogenetic study of the prevail STs in China, including ST9, ST8, ST121, and ST87, and each corresponding STs isolates from other countries, revealed the distribution of our studied isolates in the context of global isolates. ST9 *L. monocytogenes* in this study were grouped into several clusters throughout the phylogenetic tree of isolates from around the world, indicating that this clone from food in China was genetically diverse. ST9 was a prevalent clone isolated from foods, especially from meat products, in many countries (Martín et al., 2014; Maury et al., 2019; Wang et al., 2021). Different genetic characteristics of ST9 *L. monocytogenes* were spread and circulated globally with the frequent international trade of food. The majority of *L. monocytogenes* isolates of ST8 and ST121 from China were both grouped into two clusters, while isolates of these two STs from other countries were distributed in different clusters (Figures 3B,C). It indicated that ST8 and ST121 isolates from China were more closely related to another among each cluster, and showed heterogeneous with isolates from other countries. The possible reason is that these two STs are adapted to different food niches in China and other countries. The results of the phylogenetic analysis of ST87 *L. monocytogenes* in this study are consistent with that of our previous comprehensive and in-depth study of this clone in China (Wang et al., 2019). The isolates of ST87 *L. monocytogenes* were divided into multiple sub-clusters that were divided from the root, and minor isolates from other countries were distributed among the tree without region-clustering except for four clustered United States isolates that were from environments in the same year (2017). Overall, ST-specific core-SNP analysis well revealed the genome-level diversity of clonal groups which were prevalent in China, providing unprecedented discrimination to distinguish isolates within certain clonal groups.

Due to the use of several stress-adaptive mechanisms to withstand a wide variety of stressful conditions, *L. monocytogenes* isolates are able to colonize in different ecological niches. SSI-1 contributes to *L. monocytogenes* adapting to low pH and high salt concentration, while SSI-2 helps *L. monocytogenes* survival in alkaline and oxidative stress conditions (Ryan et al., 2010). In this study, 72.99% of lineage II isolates carried SSI-1, which included all ST8 and ST9 isolates. While all the ST121 isolates belonging to lineage II carried SSI-2. These findings gave further support for lineage II isolates being more prevalent in foods and food-associated environments than lineage I isolates. Interestingly, ST299 isolates that belonged to the rare lineage III and serogroup L were identified to overrepresent in aquatic products, which is similar to the results reported by Chen et al. (2018). It has been proposed that the ST299 have a specific ecological niche associated with aquatic products and the environment, while our study found that all ST299 isolates carried SSI-1. However, the contribution of SSI-1 in adapting to aquatic ecological niches in ST299 *L. monocytogenes* requires further functional confirmation. In addition, LGI-2 carried cadmium resistance cassette and arsenic resistance cassette which were associated with adaptation to environment, and was found a propensity with hypervirulent serogroup IVb clones, suggesting possible associations with *L. monocytogenes* virulence (Parsons et al., 2020). A variant of genomic island LGI3 lacking *cadA1C* cassette was found in all ST101/CC101 isolates in this study, which was the same with the observation of the study of Gray et al. (2021). However, Palma et al. (2020) found that the remaining 25 genes of LGI3 were significantly enriched in the persistent CC101 clone from the smoked-fish plant, indicating that this variant of LGI3 still contributes to persistent contamination of the ST101/CC101 clone.

As an important foodborne pathogen, several virulence potentials ultimately contribute to the ability of *L. monocytogenes* to infect humans. Except for species-specific LIPI-1, both LIPI-3, and LIPI-4 were found in a subset of lineage I *L. monocytogenes* isolates that were overrepresented in human infection cases. Strikingly, one ST11 strain (ICDC-LM277) of lineage II was isolated from raw meat and harbored LIPI-3. The LIPI-3 was also found in the genomes of eight ST11 *L. monocytogenes* isolates which caused an outbreak of listeriosis manifesting febrile gastroenteritis in Italy in 2016 (BioProject accession no.: PRJNA436467; Maurella et al., 2018). It suggested that ST11 *L. monocytogenes* isolates carrying LIPI-3 might have an increased risk of causing gastrointestinal infection in humans. LIPI-4, which is associated with increased neural and placental tropisms of *L. monocytogenes*, was found in all ST87/CC87 and ST296/CC88 isolates in this study. ST87 had been recognized as the dominant ST in clinical listeriosis in China, particularly associated with maternal-neonatal and central nervous system infections (Wang et al., 2019). Intact InlA is more favorable for *L. monocytogenes* to cross the intestinal barrier and establish a systemic infection, 70.19% (226/322) isolates in this study have full length of InlA, while 98.61% (71/72) ST9 isolates have truncated InlA, the under representation of the ST9 isolates in human clinical cases may be due to the frequent occurrence of PMSC mutations.

*L. monocytogenes* has been present sensitive to most antibiotics. For the treatment of listeriosis, ampicillin and penicillin-based agent is effective against this bacterium (Fischer et al., 2020). In present study, aminoglycosides resistance gene *aacA4* was exclusively found in a subset of ST3 *L. monocytogenes* isolates, consistent with data obtained from Brazil and Poland (Camargo et al., 2019; Kurpas et al., 2020). The *aacA4* gene was carried by a 57-kb prophage, and it is strongly associated with ST, but not with isolation location or type of food source. The standard therapy for listeriosis usually combined with gentamicin (aminoglycoside antibiotic; Fischer et al., 2020), monitoring *aacA4* gene in *L. monocytogenes* isolates especially ST3 strains is important for listeriosis clinical treatment. Tetracycline resistance was the most common phenotype in food source *L. monocytogenes* in China (Yan et al., 2019), and we found that 4.3% of isolates carried the tetracycline resistance genes *tetM* or *tetS* in this study, and the *tetM* gene is associated with Tn916 and the *tetS* gene is carried by a plasmid, which was consistent with that of previous studies (Bertsch et al., 2013; Haubert et al., 2016). The high genetic identity of *tetM*-containing transposons between *L. monocytogenes* and *B. subtilis* or *E. faecalis* suggests that horizontal transfer of antibiotic genes can occur between different genera of bacteria in the same niche. In addition, the 18-kb Tn916 transposon could be found in both lineage I and lineage II isolates. It is a warning that transposon cannot be ignored for its role in the dissemination of the resistance genes across the lineages in *L. monocytogenes*. However, no evidence showed the harboring of resistance genes was related to isolation location, isolation year and the type of food source.

## Conclusion

In this study, 322 *L. monocytogenes* isolates were selected and sequenced to characterize the population structure of food source isolates in China. Serogroup IIa and ST9 were identified as the most prevalent serogroup and ST, respectively. Core-SNP analysis classified all the isolates into different clusters that corresponded to the different clonal complexes which were further grouped into three lineages. In-depth core-SNP analysis based on specific CC or ST reveals the potential epidemiological relationship of isolates in China: (i) ST122 clone might diverge from ST9 clone; (ii) persistent contamination, cross-contamination, and transmission from a single source to different markets were found in markets of China; (iii) the prevalent subtypes ST8 and ST121 were heterogeneous with worldwide isolates. Genomic analysis identified LIPI-3 in lineage II (ST11/CC11) isolates and a novel PMSC type in *inlA* gene, which increased the understanding of the genomic diversity of *L. monocytogenes*.

## Data availability statement

The datasets presented in this study are deposited in the China National Microbiology Data Center (NMDC), with accession number NMDC60016487 to NMDC60016827 (https://nmdc.cn/resource/genomics/genome/).

## Author contributions

SJ, CY, and YaW conceived and designed the research study. SJ, YiW, LLi, and PM performed the sample collection and DNA extraction. ZS and SJ analyzed the data. SJ, LLu, CY, and YaW write and revise the manuscript. All authors have read and approved it for publication.

## Funding

This work was supported by grants from National Key R&D Program of China (2018YFC1603800); National Institute for Communicable Disease Control and Prevention, China CDC (2021ZZKT003, 131031102000210003-07007); National Natural Science Foundation of China (31800004).

## Conflict of interest

The authors declare that the research was conducted in the absence of any commercial or financial relationships that could be construed as a potential conflict of interest.

## Publisher’s note

All claims expressed in this article are solely those of the authors and do not necessarily represent those of their affiliated organizations, or those of the publisher, the editors and the reviewers. Any product that may be evaluated in this article, or claim that may be made by its manufacturer, is not guaranteed or endorsed by the publisher.

## Footnotes

^1^ https://bigsdb.pasteur.fr/listeria

^2^ https://itol.embl.de/

^3^ http://jameshadfield.github.io/phandango/
