# Supplementary figures and images for: Whole-genome sequencing reveals genomic characterization of Listeria monocytogenes from food in China
Source: Front Microbiol. 2023 Jan 16;13:1049843. doi: 10.3389/fmicb.2022.1049843 (PMC9885130; doi:10.3389/fmicb.2022.1049843)

A

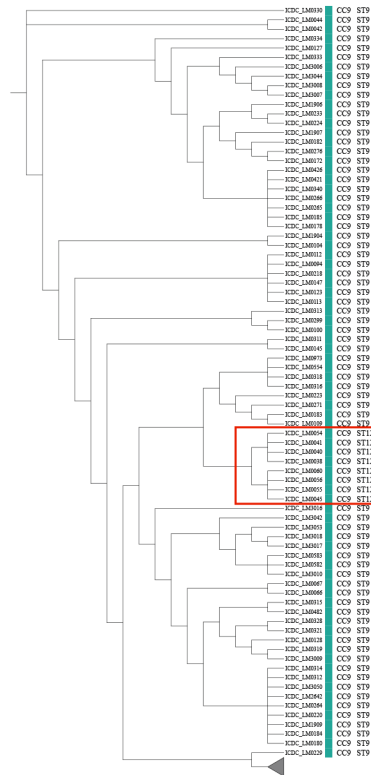

B

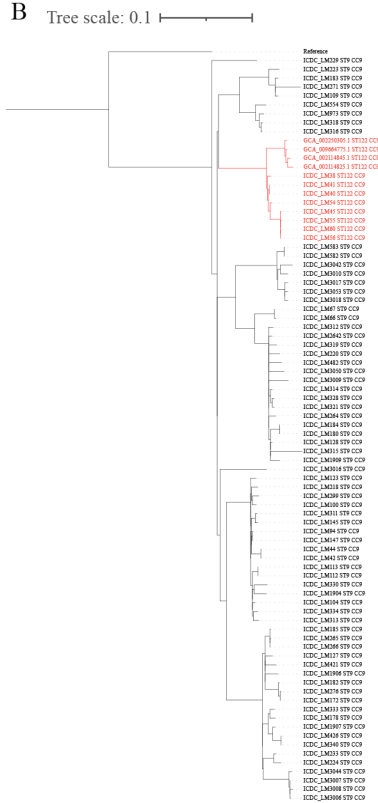

C

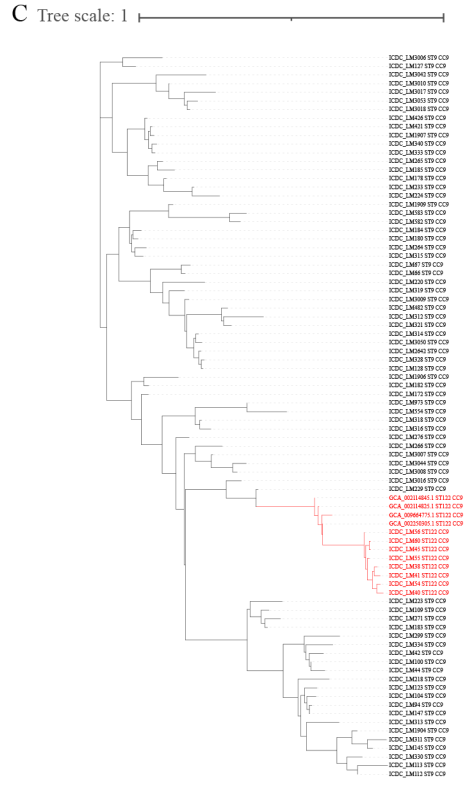

Supplement: Supplementary file 4 [file Data_Sheet_1.PDF]
